# Apraxic agraphia: An insight into the writing disturbances of posterior aphasias

**DOI:** 10.4103/0972-2327.53082

**Published:** 2009

**Authors:** Gopee Krishnan, Soorya Narayana Rao, Bellur Rajashekar

**Affiliations:** Department of Speech and Hearing, Kasturba Hospital, Manipal University, India; 1 Department of Neurology, Kasturba Hospital, Manipal University, India

**Keywords:** Apraxic agraphia, aphasia, pure motor agraphia

## Abstract

**Background:**

Reading and writing disturbances are common accompaniments of aphasia following brain damage. However, impaired writing in the absence of apparent primary linguistic disturbances is infrequently reported in the literature.

**Materials and Methods:**

A 67-year-old right-handed subject underwent neurological, neuroradiological, and linguistic investigations following development of a minimal right upper limb weakness.

**Result:**

The patient had polycythemia and the neurological investigation revealed right upper limb paresis. The neuroradiological investigation revealed hypodense areas involving the gray-white matter of the left postero-parietal and frontal lobe, left caudate and lentiform nuclei, and the anterior limb of the internal capsule, suggesting an infarct. The linguistic investigation revealed a mild anomic aphasia with apraxic agraphia. This mild anomic aphasia resulted primarily from the relatively poor scores on the verbal fluency tests.

**Discussion:**

The marked writing impairment, even with the left hand, points to disturbances in written output – apraxic agraphia – in the presence of near-normal spoken output. This finding should raise suspicion about hidden apraxic agraphia in subjects with posterior aphasias.

## Introduction

Agraphia refers to an acquired loss/impairment in writing following brain damage. As with the alexias, there are different types of agraphias reported in the literature: For example, phonological agraphia,[1] lexical agraphia,[2] and semantic agraphia.[3] Each of them stems from deficits at various levels of the cognitive processes involved in writing. However, the occurrence of selective difficulty in writing (pure agraphia)—with sparing of speaking, understanding, and reading—has been only infrequently reported in the literature.[4]

As early as the 19^th^ century, Pitres[5] defined pure motor agraphia as ‘the aphasia of hand.’ In the recent literature, pure motor agraphia has been referred to as ‘apraxic agraphia.’ It is characterized by deteriorated orthographic production with otherwise normal praxis, sensory/motor functions, and preserved oral spelling and typing.[6] Patients with apraxic agraphia show normal oral spelling skills despite having marked difficulty in written performance.[4] Friedman and Alexander[7] reported that pure motor agraphia involved problems in accessing the appropriate allographic codes and/or selecting the appropriate program specifying the movements needed to form letters. Pure motor agraphia could also result from incorrect letter selection, production of poorly formed letters, inability to maintain a particular script or case consistently, and a variable range of difficulty in copying letters or words.[8,9]

There is little consensus on the site of the lesion causing apraxic agraphia. Some authors have postulated that the lesion is in the left parietal lobe,[6,7,10] whereas others have suggested that the frontal lobe is the site of the lesion.[11,12] In addition to this, there have also been reports of subcortical (thalamic) lesions leading to apraxic agraphia. For instance, Ohno *et al*.[13] have reported a subject with apraxic agraphia following left thalamic infarction. However, these authors attributed the impaired writing performance to the cortical hypoperfusion in the dorsolateral prefrontal cortex that was evident from the positron emission tomography (PET) study.

Very recently, Mariën and colleagues[14] reported a 72-year-old right-handed subject with apraxic agraphia following right cerebellar damage. However, their subject, in addition to the agraphia also revealed mild aphasia. The authors hypothesized the presence of a crossed cerebellocerebral diaschisis as the possible mechanism of agraphia in their subject.

It is thus evident from the available though limited reports that writing skills can be impaired independently and disproportionately with respect to other language skills. However, the lack of consensus on the site of the lesion leading to apraxic agraphia complicates the scenario. Does a posterior brain lesion lead to apraxic agraphia? Can we expect elements of apraxic agraphia in aphasic agraphia? We address these questions in this study.

## Case Report

A 67-year-old, right-handed subject was brought to the Department of Neurology, Kasturba Hospital, Manipal, India, in October 2007 following development of weakness of the right upper limb. He was a retired lecturer of economics by profession. The weakness of right upper limb had been noticed for 15 days prior to his admission to hospital.

The patient admitted to having reduced motivation to speak for two months prior to the hemiparesis.Premorbidly, he was reported to be highly talkative and active among his circle of friends. The neurological evaluation revealed right upper limb paresis. Hematological examination revealed polycythemia.

**Figure 1:**
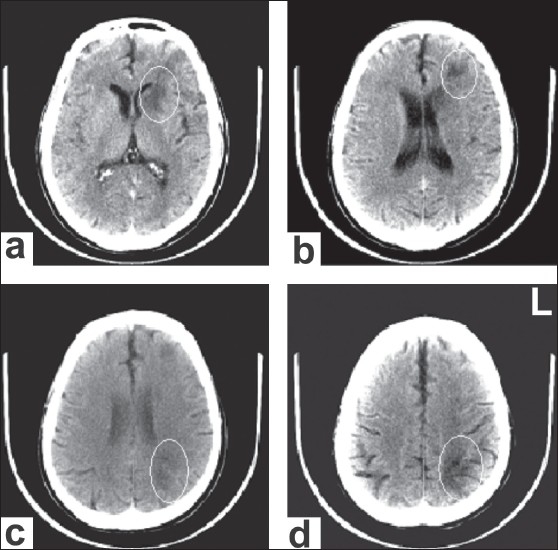
Plain axial CT showing hypodensity in the gray-white matter region of the left posterior parietal and frontal lobe, left caudate and lentiform nucleus, and the anterior limb of the internal capsule

The plain CT study performed on the following day of admission revealed hypodense areas involving the gray-white matter of the left postero-parietal and frontal lobe, the left caudate and lentiform nuclei, and the anterior limb of the internal capsule; these findings were suggestive of an infarct [Figure 1].

Mr. K was evaluated on his language skills using the Kannada version of Western Aphasia Battery (WAB). He performed fairly well on all subtests of WAB, except for showing subtle impairment in the naming skills. The following are the aphasia quotients (AQ) on various subtests of WAB (out of a maximum AQ of 10 for each subtest): Fluency: 10, auditory verbal comprehension: 9.5, repetition: 10, and naming: 7.6; these scores suggested a diagnosis of minimal anomic aphasia.

He could read even lengthy paragraphs without any pronunciation errors or hesitations and could readily comprehend what had been read. It was also noticed that during the period of hospitalization, Mr. K spent most of his time reading newspapers and magazines.

**Figure 2:**
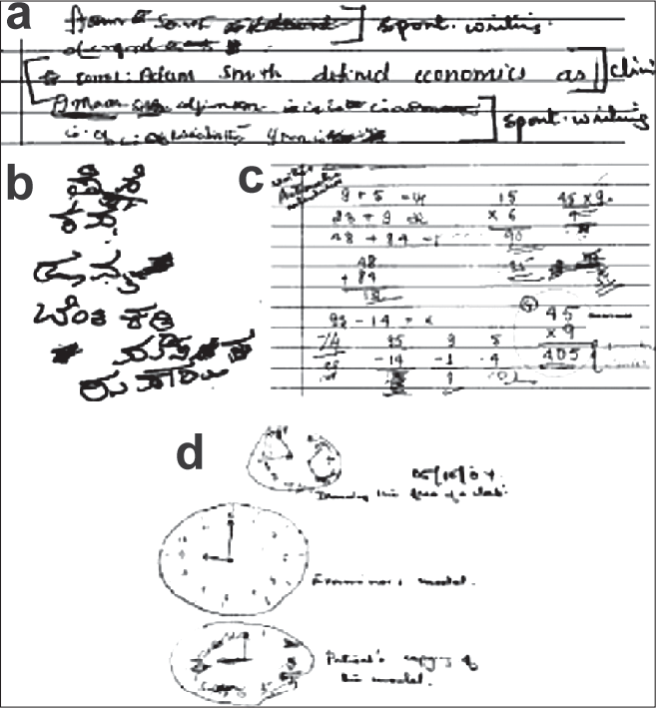
Mr. K's performance on various tasks. (a) spontaneous writing in English; (b) spontaneous writing in Kannada (mother tongue); (c) attempts at written arithmetical calculations; and (d) attempts to draw the face of the clock showing 9 o'clock

For the assessment of his writing skills he was asked to write a passage on a topic of his interest [Figure 2a and b]. He failed miserably in this task. At the time of this investigation, his motor power (4/5, as examined by an experienced neurologist, the second author) was returning to the normal level; however, the writing was highly illegible, with poorly formed letter shapes and orientation. In addition to the obviously poor handwriting, the written content was poorly formed. We then asked the subject to write with his left hand. However, he performed badly, with both written content and legibility being very poor; the poor legibility was of course expected with left hand usage. Attempts to copy a sentence also revealed his spontaneous writing impairments. However, he exhibited normal recognition of letters when the examiner named them.

In addition to the writing difficulty, Mr. K exhibited calculation difficulties [Figure 2c]. However, single-digit and simple two-digit mental calculations were relatively preserved. His impairments were obvious during the attempts to solve problems requiring multiplication and division tasks.

In order to assess his cognitive skills, the Kannada version of Addenbrook's Cognitive Examination (under preparation) was administered. Mr. K performed fairly well on all the subtests except on the visuospatial skills. When asked to draw the face of a clock showing 9 o'clock he had apparent difficulty [Figure 2d].

## Discussion

The process of writing consists of the translation of allographic symbols – the representation of letter shapes – into writing movements.[15] Mr. K exhibited apparent difficulties in this task despite his near-normal performance in other modalities of language usage. The neuroimaging investigation showed two areas of hypodensity: One in the left superior parietal lobe and the other in the left frontal cortico-subcortical areas. Though the chronological sequence of these two lesions was difficult to infer from the images, it is evident from the case history that the right upper limb weakness began 15 days prior to the hospitalization.

In our subject, it is rather difficult to attribute the impaired writing to the posterior brain damage. However, it is possible to deduce it. If the agraphia were due to the anterior brain damage, then it should have been limited to the right hand. However, Mr. K showed impaired writing (poor legibility as well as impaired written sentence formulation) even with the left hand, thereby ruling out the possibility of the anterior lesion being the cause of the presenting agraphia. It has been known for quite long that left parietal lobe damage could lead to bilateral apraxia.[16] Moreover, the additional difficulties such as impaired calculation and visuospatial skills – some common accompaniments of aphasic agraphia following a left parietal lobe lesion – are apparent in our subject, suggesting the possible contribution of left parietal lobe damage to the apraxic agraphia.

The selective impairment in writing, with preservation of the rest of the language input and output modalities at normal or near-normal levels, has important implications on our understanding of language processing in the brain. The dissociation between writing and the other language skills is evidence of the presence of a specific neuroanatomical substrate for writing. The normal recognition of letter shapes showed that Mr. K had intact visual imagery of letters. In the process of writing, one retrieves these mental imageries of letters and translates them into written output. In much the same way as occurs in verbal apraxia in anterior lesions, the subject had extreme difficulty in translating these imageries into written form. The impaired written sentence formulation could parallel impaired sentence production in a pure verbal apraxic subject (without non-fluent aphasia). Various forms of aphasic agraphias reported elsewhere in the literature are due to more central impairment in language processing. That is, the underlying aphasia in such conditions could result in (in addition to the core deficits in comprehension and/or expression) disturbances in reading as well as in writing. Quite distinct from this, apraxic agraphia (pure motor agraphia) results in impaired written language output.

It is therefore evident from Mr. K's case that writing could be impaired at the output level, despite normal central language processing and absence of peripheral motor impairment such as hemiparesis (as evidenced by his impaired writing content with the intact left hand). The incidence of apraxic agraphia is rather low compared to that of verbal apraxia. This casts doubts about the possibility of apraxic agraphia occurring in posterior brain damage. Posterior aphasias are almost always associated with reading and writing impairments. Aphasic agraphia associated with posterior brain damage, at least in principle, does not necessarily lead to poor letter legibility. Hence, the presence of illegible handwriting in posterior aphasia may be viewed as a possible indicator of a ‘hidden’ apraxic agraphia. Quite often, the writing disturbances in posterior/fluent aphasias have been considered as true aphasic agraphias. However, the evidence from Mr. K reveals that even in the absence of apparent (fluent) aphasia, the written output could be compromised. Put another way, subjects with a posterior dominant temporoparietal lesion may exhibit not only aphasic agraphia but also apraxic agraphia. That is, the writing disturbances seen in such subjects may be a combination of both aphasic and apraxic agraphias, with the latter often being masked by the aphasic manifestation of the subject. A simple logical step to isolate apraxic from aphasic agraphia would be to examine the legibility of the letters. In pure posterior aphasic agraphia, there is no reason to expect any poorly formed letter shapes, though the written output would be empty (similar to the spoken output). Therefore, the presence of illegible letters should raise the suspicion of a hidden apraxic agraphia in subjects with fluent aphasia. This undoubtedly has important implications from the rehabilitative perspective.

## Summary

The writing disturbances (poor legibility and written content) in subjects with posterior brain lesions may be due to a combination of an overt aphasic agraphia and a covert apraxic agraphia. The aphasic agraphia may be associated with classical expression and/or comprehension deficit(s). Apraxic agraphia shows impairment only in the written output modality, sparing the rest of the modalities. This claim, however, remains speculative, and confirmation requires observations from a larger number of subjects. Awareness that aphasic agraphia can coexist with hidden apraxic agraphia helps the clinician to chalk out an effective intervention plan for subjects with posterior brain damage.
